# Supplementary material for: The Consequences of Water Interactions with Nitrogen-Containing Carbonaceous Quantum Dots—The Mechanistic Studies
Source: Int J Mol Sci. 2022 Nov 18;23(22):14292. doi: 10.3390/ijms232214292 (PMC9694419; doi:10.3390/ijms232214292)
Supplement: Supplementary file 1 [file ijms-23-14292-s001.zip › ijms-2032003-Supplementary.pdf]

Supporting Materials

The Consequences of Water Interactions with Nitrogen-Containing Carbonaceous Quantum Dots—The Mechanistic Studies

Marek Wiśniewski

Physicochemistry of Carbon Materials Research Group, Faculty of Chemistry, Nicolaus Copernicus University in Toruń, Gagarina 7, 87-100 Toruń, Poland; marekw@umk.pl

The simplest and most popular form of the original DW equation is the sum of Langmuir (the adsorption on primary sites) and DS1 isotherms (describing the adsorption on secondary sites available for water molecules):

$$a = a_{prim} + a_{\sec} = \underbrace{\frac{a_{mL} K_L h}{1/p_s + K_L h}}_{L} + \underbrace{\frac{a_0 ch}{1 - ch}}_{DS1} \qquad \text{(S1)}$$

where a_{mL} is the total surface concentration of all Langmuir-type (high-energy) sites, K_L is the Langmuir constant, $h = p/p_s$, a_0, and c are the number of adsorption centers and the kinetic constant related to the adsorption on secondary sites (of Dubinin–Serpinsky type), respectively.

It is well-known that the difference in the shape of water isotherms is caused by the combined effects of surface centers energies: the higher – primary and the lower – secondary.

Water is known to have an extremely low affinity toward the 'pure' benzene ring and a high affinity toward polar sites leading even to chemisorption. Subsequently, those adsorbed molecules on primary sites (the mentioned above types of centers should be considered) can become secondary ones and adsorption on them occurs following the original Dubinin-

Serpinsky mechanism. Thus, the total adsorption is the sum of the adsorption on all, available for water molecules, primary (a_{prim}) and secondary sites (a_{sec}).

This simple model was presented as applicable to the description of different adsorption data [see, e.g. 39, 40].

As N-CQDs possess a large amount of high-energy adsorptive centers for the fitting of experimental adsorption data (Fig. 3) we have chosen the D-W equation (S1). It is seen that it fits the experimental data satisfactorily – the determination coefficient is 0.9992, the standard error of estimates is low for all fitted parameters (Tab.S1). The Langmuir constant K_L is related to the affinity between the adsorbate and the adsorbent, while the parameter *c* is the ratio of adsorption and desorption constants; the value of $c = 0.89$ suggests highly-interacting sites.
In Tab.1 also the components of Eq.(S1) are shown. It is seen that practically in the p/p_s range 0-0.008 the Langmuir term is sufficient to fit the data correctly, the DS1 term is needed already for $p/p_s > 0.01$.

We get a_{mL} equal 8.95 mol H_2O/g(N-CQD), which is reached at a pressure below 0.01 p/p_s where H_2O was adsorbed mainly on primary sites. Products of such chemisorption formed the secondary sites (a_0 = 11.99 mmol/g).

Table.S1 The estimates of DW model parameters.

Parameter	Estimate	Std. error
a_{mL} [mmol/g]	8.95	0.0963
K_L	295.64	3.3099
C	0.89	0.0018
a_0 [mmol/g]	11.99	0.1718
R^2	0.9992	

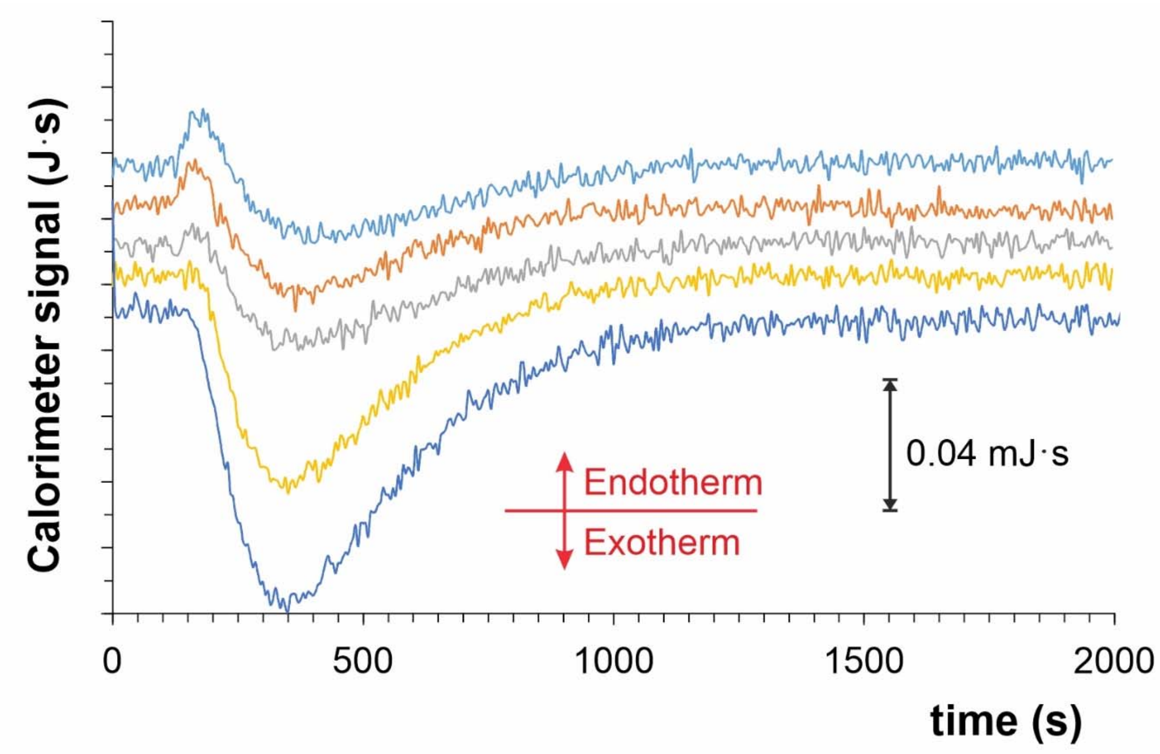

Figure S1. Experimental calorimetric data for the first five points showing endoenergetic effect at the beginning of the isotherm.

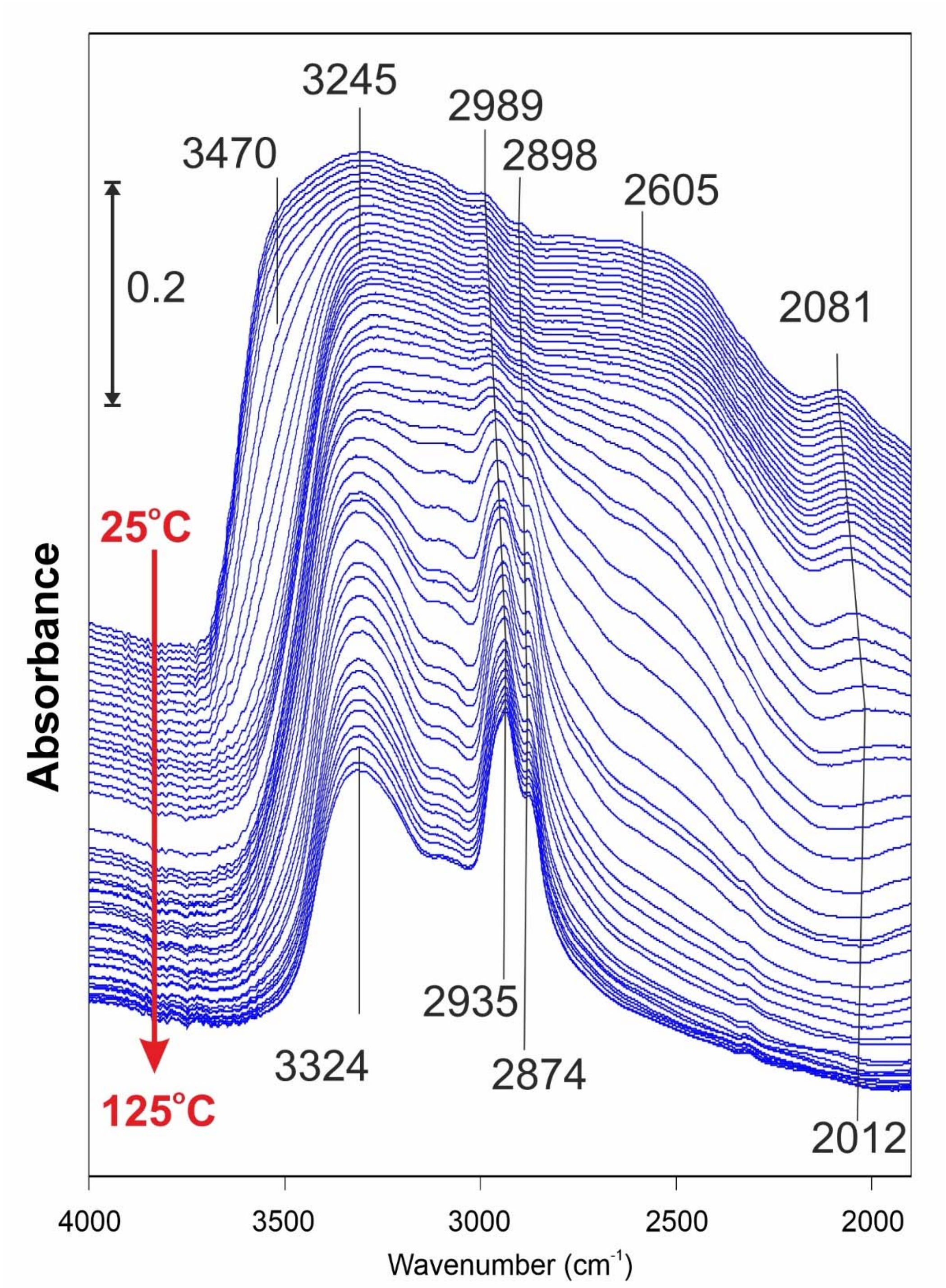

Figure S2. The OH stretching spectral region during isobar performing. Note spectra were taken each 2°C.

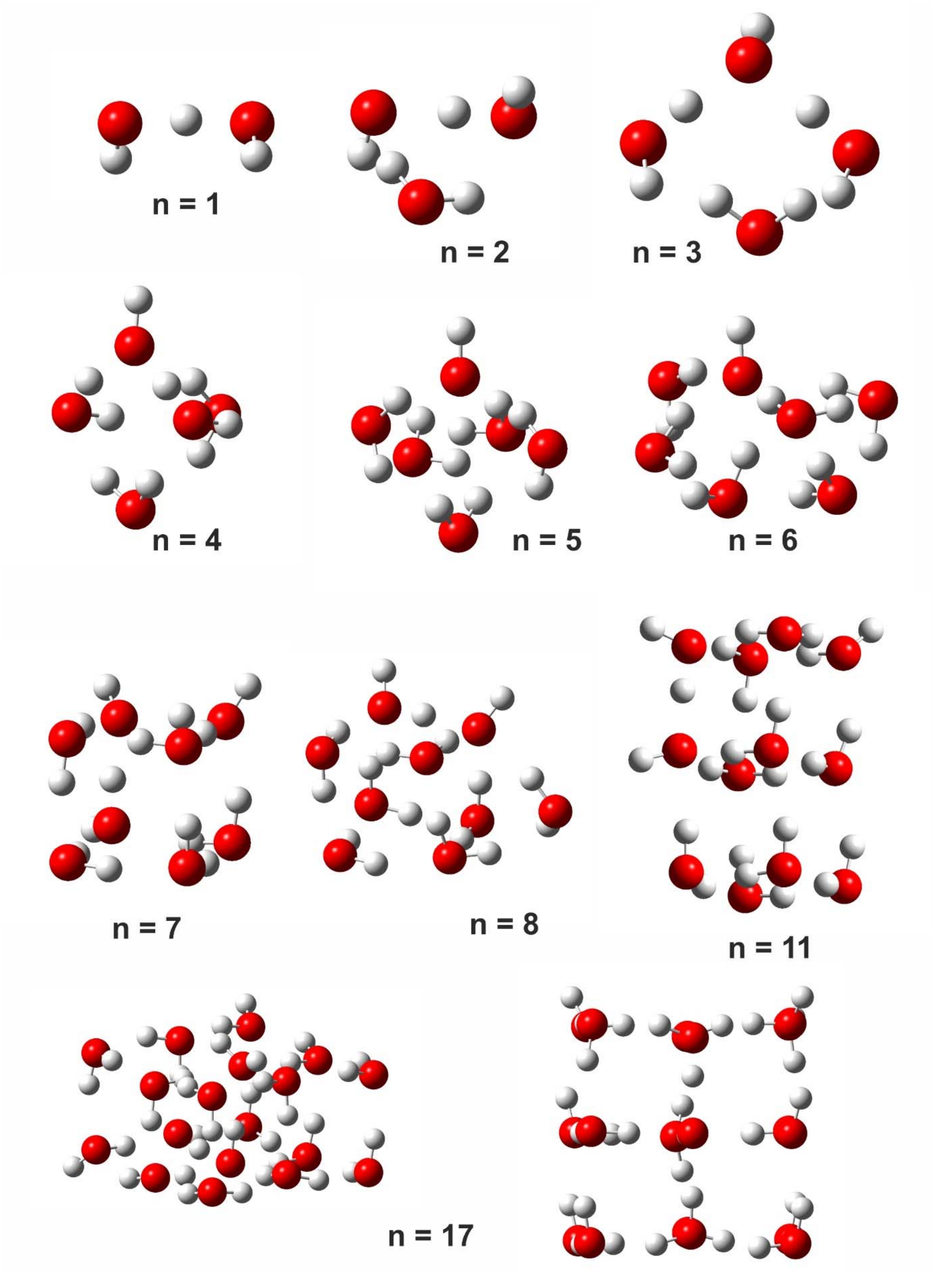

Figure S3. Modeled structures of $[OH(H_2O)_n]^-$ (n = 1 – 17).

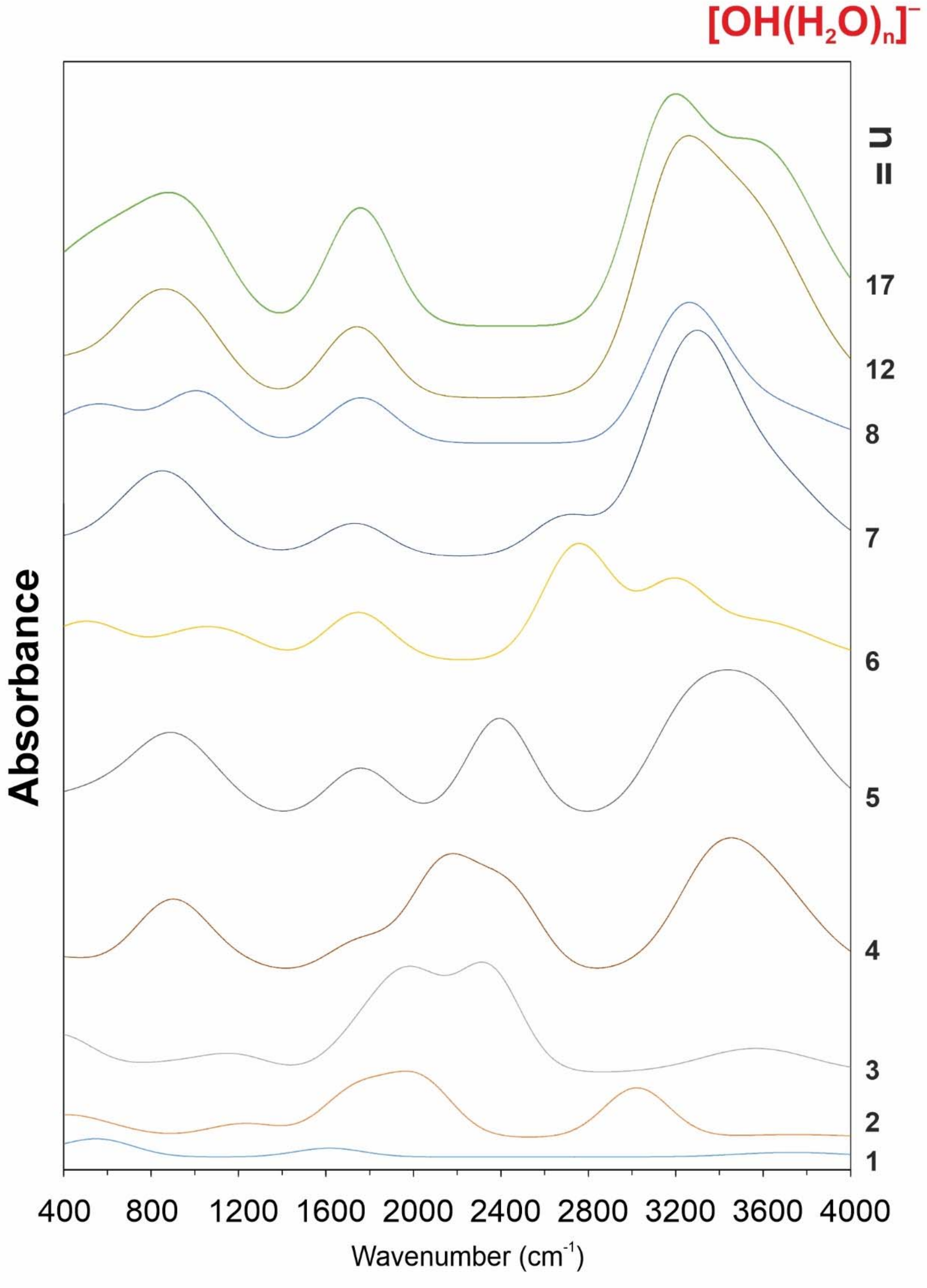

Figure S4. Simulated FTIR spectra of modeled $[OH(H_2O)_n]^-$ (n = 1 – 17) structures.

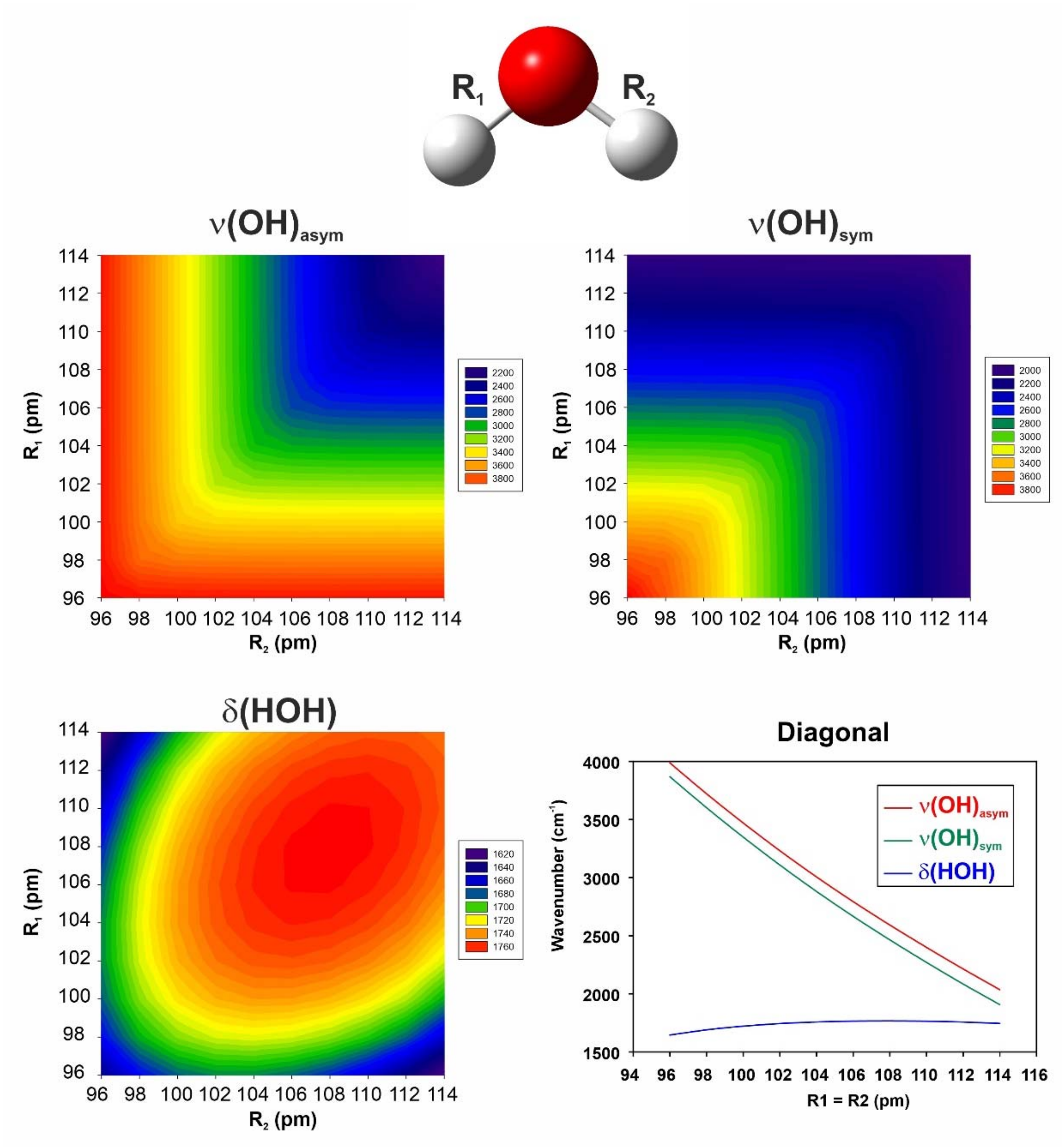

Figure S5. The effect of R_1 and R_2 distance on H_2O IR bands position changes.

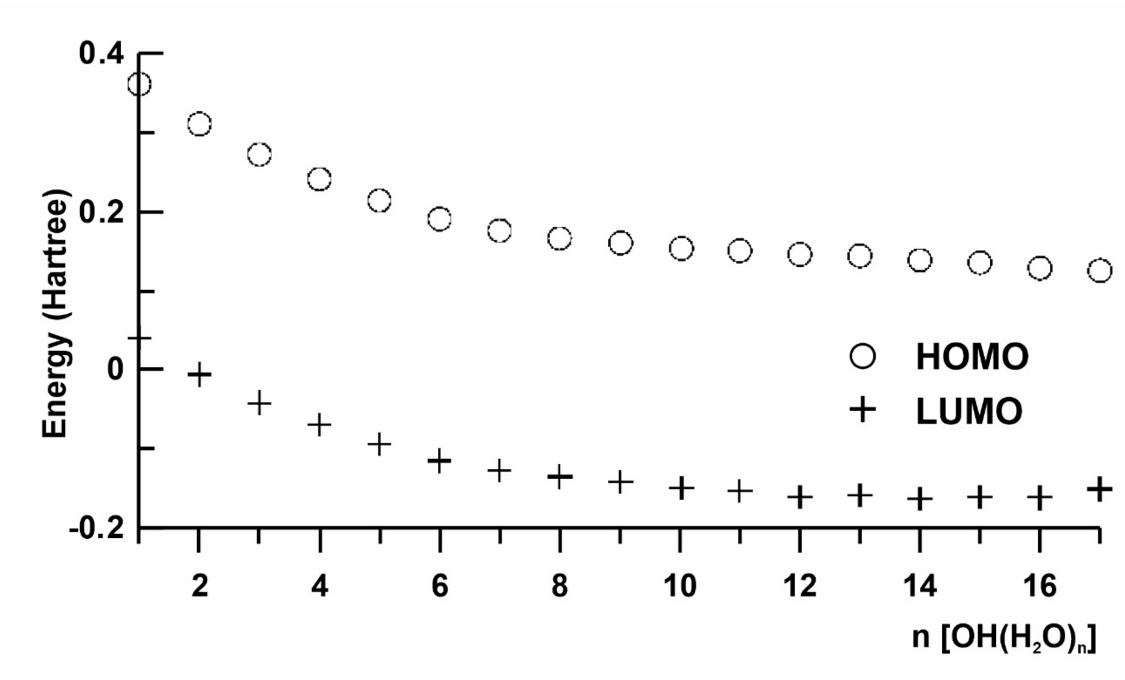

Figure S6. Orbital HOMO and LUMO energies changes as a function of n $[OH(H_2O)_n]^-$ (n = 1 – 17).

Note that the Koopmans' theorem [41] in terms of HOMO and LUMO energies is not satisfied in this case (Fig.S6). By treating the IP and EA difference as natural bandgap energy, one can conclude that smaller clusters (n→1) tend to the higher isolating nature of $[OH(H_2O)_n]^-$.

Materials and methods

Nitrogen-enriched carbon quantum dots synthesis

Nitrogen-enriched carbon quantum dots (N-CQDs) were prepared via hydrothermal carbonization of gelatine at 200 °C for 6 h, under 19 MPa. After cooling down to room temperature, the obtained brown solution (N-CQDs suspension) was ready for further examination. We have compared the fluorescence spectra for the as-obtained sample and two others subjected to further purification processes (centrifugation at 15 000 g for 30 min. and dialysis).[8,27]

N-CQD physicochemical characteristics

The full physicochemical characterization was performed and described recently.[8,27] These analyses concern: Elemental analysis of prepared N-CQD samples was performed by energy-dispersive X-ray method (EDX). The equipment used for the EDX analysis was LEO Electron Microscopy Ltd, England, model 1430 VP. The High-Resolution Transmission Electron Microscopy (HRTEM) images were taken using a transmission electron microscope F20X-TWIN (FEI-Tecnai) operated at 200 kV. The drop of sample solution was placed on a Cu-grid coated with an ultrathin amorphous carbon film and then dried under ambient conditions. (Fig. S7) The morphology of N-CQD dispersed on Si wafers was analyzed at room temperature in the air using a microscope with a scanning SPM probe of the NanoScope MultiMode type (Veeco Metrology, Inc., USA), which operated in a tapping mode. Here are presented the SEM pictures of N-CQD immobilized on glass beads. The fluorescence spectra were measured with a fluorescence spectrometer RF-5001PC (Shimadzu, Japan). The excitation maximum was experimentally established at 360 nm. UV-vis spectra of N-CQDs were acquired using Jasco 660 spectrometer in a range of 200 – 800 nm.

FTIR analysis

Fourier-transform infrared (FTIR) spectroscopy data were acquired by using a Vertex V70 (Bruker Optic), in ATR mode techniques (single reflection using diamond crystal), in the frequency range 6000–15 cm^{-1}.

For isobars experiments, transmission mode was used. The sample sprayed on Si-wafer was dried at 25°C under vacuum. Next, water vapor (30 mBa) was added. After the equilibrium was reached, the temperature raised up to 125°C with the ramp 2°C/min

Calorimetric analysis

Water vapor adsorption isotherms were measured volumetrically at 298 K and differential enthalpy of adsorption (q^{diff}) was determined using a Tian–Calvet isothermal microcalorimeter constructed in our laboratory.[37] The related molar entropy changes of the adsorbed molecules (ΔS) was calculated using the basic thermodynamic relations (eq.S2);

$$\Delta S = \frac{q^{diff}}{T} + R \ln \frac{p}{p_s} + R \qquad \text{(S2)}$$

where R is the gas constant, and p and p_s are the equilibrium and saturated vapor pressures, respectively.

To perform this experiment known mass of N-CQD samples was put on glass bead support Figs. S8.

Ab-initio studies

All calculations (at B3LYP levels of theory using 6–311G(d,p) basis sets) were performed using geometries optimized for the parent compounds as shown in Figure S2 using Gaussian '09 program.[38] For the calculation of the ionization potentials (IP) and electron affinities (EA) for systems with N, N – 1, and N + 1 electrons (for the same geometry) were calculated. Thus, the reactivity of these model layers is discussed based on the global reactivity parameters as IP, EA, global hardness (η).

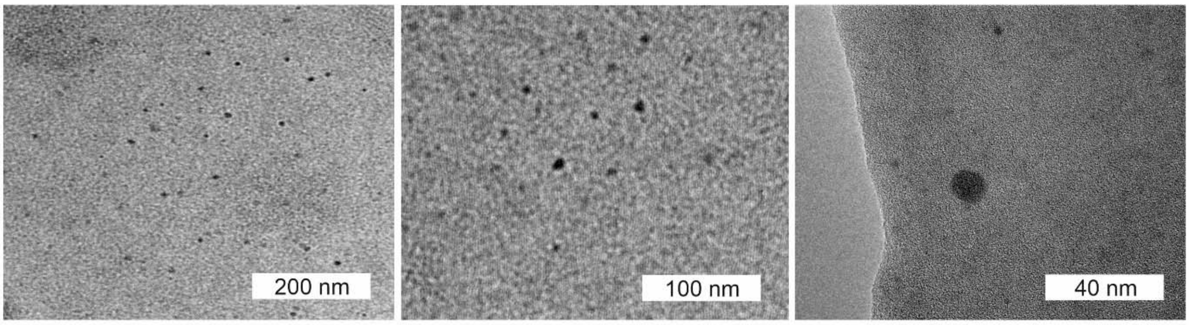

Figure S7. HRTEM pictures of synthesized N-CQD

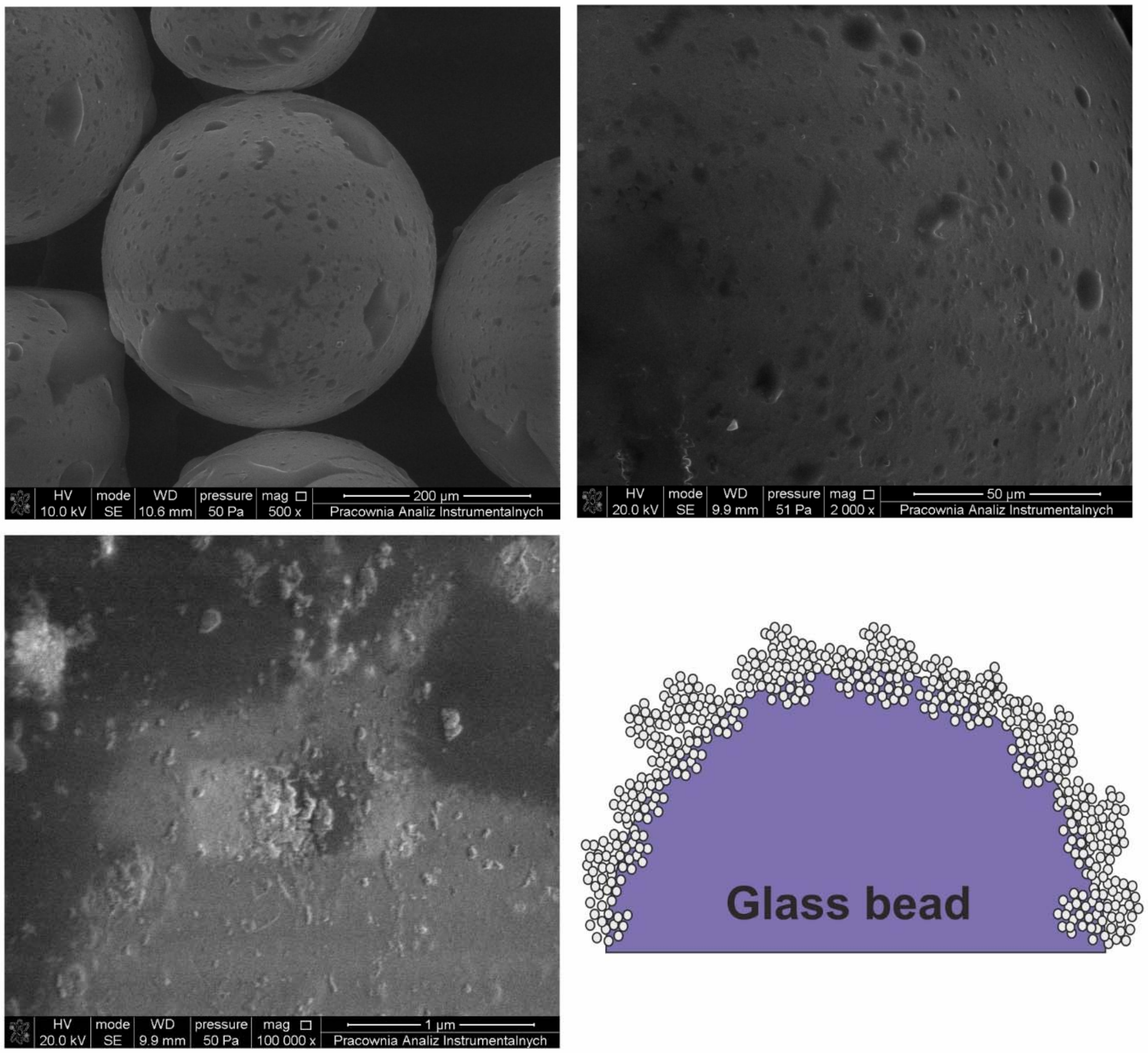

Figure S8. SEM pictures proving N-CQD successful immobilization on glass beads.
